# FourierDrug: a domain generalization framework for robust drug response prediction via frequency-space asymmetric attention

**DOI:** 10.1093/bioinformatics/btag276

**Published:** 2026-05-05

**Authors:** Ran Song, Yinpu Bai, Xuejun Liu, Hui Liu

**Affiliations:** College of Computer and Information Engineering, Nanjing Tech University, Nanjing, Jiangsu 211816, China; College of Computer and Information Engineering, Nanjing Tech University, Nanjing, Jiangsu 211816, China; College of Computer and Information Engineering, Nanjing Tech University, Nanjing, Jiangsu 211816, China; College of Computer and Information Engineering, Nanjing Tech University, Nanjing, Jiangsu 211816, China

## Abstract

**Motivation:**

Accurate prediction of drug response remains a major challenge in precision oncology, particularly at the single-cell level and in clinical settings, due to significant distribution shifts between preclinical models and real-world patient data. Existing approaches often rely on transfer learning from cell lines to target domains, but typically require access to target-domain data during training, which is frequently unavailable in practice.

**Results:**

We propose FourierDrug, a novel domain generalization framework for robust drug response prediction. Given gene expression profiles, the model performs Fourier transformation to project features into the frequency domain and introduces an asymmetric attention mechanism that encourages drug-sensitive samples to form compact clusters while driving resistant samples to be more dispersed. This design facilitates the learning of domain-invariant yet task-relevant representations. Extensive experiments demonstrate that FourierDrug effectively leverages diverse source domains and generalizes well to unseen cancer types. Notably, when evaluated on single-cell and patient-level prediction tasks, our method—trained solely on in vitro cell line data without access to target-domain data—consistently outperforms or matches state-of-the-art approaches.

**Availability and implementation:**

The source code and processed datasets are available at: https://github.com/hliulab/FourierDrug.

## 1 Introduction

To study the drug responses of *in vitro* cancer cells, several projects have utilized high-throughput profiling to assess cell viability when subjected to varying drug concentration treatments and yielded half-maximal inhibitory concentration. For instance, the Genomics of Drug Sensitivity in Cancer project (GDSC) (Yang *et al.* 2013) has assayed the sensitivity of more than 1000 of cancer cell lines to more than 200 of compounds. The Cancer Cell Line Encyclopedia (CCLE) (Barretina *et al.* 2012) is another effort that compiles genomic, transcriptomic, and drug sensitivity data for over 1000 cancer cell lines. These public resources promote the development of machine learning methods for predicting drug response based on gene expression profiles (Ma *et al.* 2021, Chawla *et al.* 2022, He *et al.* 2022). However, while some drugs exhibit promising sensitivity against tumor cells cultured under laboratory conditions, such observations offer limited guidance for clinical drug selection due to substantial discrepancy between *in vitro* cellular context and *in vivo* physiological environment. On the other hand, the identification of drug-resistant subpopulations within tumors is a critical step in understanding treatment resistance mechanisms and guiding precision medicine. Nevertheless, drug response prediction models developed based on bulk RNA-seq data face substantial difficulties when applied to single-cell domains.

The failure of extrapolation from cell lines to single cells and clinical patients is primarily attributed to differences in data distribution (Franzén *et al.* 2019, Han *et al.* 2023). These differences are resulted from biological discrepancies between cell lines and the complex microenvironment within single cells or tumor tissues. To address this challenge, a few studies used deep transfer learning techniques to translate drug response insights derived from source domains (e.g. cell lines) to target domains (e.g. single cells or patients). For example, scDEAl (Chen *et al.* 2022) and SCAD (Zheng *et al.* 2023) utilizes domain adaptation to extract domain-invariant features between source and target domains, thereby transferring drug response knowledge from cell lines to single cells. The CODE-AE model (He *et al.* 2022) applies the domain separation network to extract shared features between cell line and patient expression profiles to predict clinical drug responses. Precily (Chawla *et al.* 2022) integrated signaling pathway and drug feature to predict drug responses *in vitro* and *in vivo*. However, existing methods predominantly rely on domain adaptation, which requires model training on predefined target domains, making them unsuitable for applications involving unseen target domains during training. In some real-world scenario, target-domain data may be currently unavailable or only accessible in future (e.g. data from newly diagnosed tumor patients).

Domain generalization (DG) is proposed to develop predictive models that can generalize to unseen test domains without access to their data during training. Recent studies in DG have achieved remarkable progress (Carlucci *et al.* 2019, Zhou *et al.* 2021, Wang *et al.* 2022) through data manipulation (Shankar *et al.* 2018, Yue *et al.* 2019, Zhou *et al.* 2021), representation learning (Ghifary *et al.* 2015, Li *et al.* 2018, Shao *et al.* 2019, Zhang *et al.* 2022, Sicilia *et al.* 2023), and meta-learning (Santoro *et al.* 2016, Finn *et al.* 2017). However, few DG method has been proposed to clinical drug-response prediction tasks. On the other hand, Fourier transform has recently been utilized as a substitute for self-attention mechanism (Lee-Thorp *et al.* 2021) or for time-series modeling (Ye *et al.* 2023, Yi *et al.* 2023, Koren and Radinsky 2024). Notably, FNet (Lee-Thorp *et al.* 2021) replaces the self-attention mechanism with Fourier transform, achieving 92% of BERT’s accuracy on the GLUE benchmark for natural language understanding tasks while delivering a 7-fold increase in training speed. FAN (Dong *et al.* 2024) demonstrated that replacing conventional neural nodes with Fourier-transform operations not only significantly reduces the number of network parameters but also markedly enhances the model’s ability to fit periodic functions. Despite these advancements, no prior work has explicitly formulated Fourier transforms and domain generalization within a unified framework for expression profile-based drug-response prediction.

In this paper, we proposed FourierDrug, a novel DG framework to generalize the predictive capacity of drug response on cell lines to out-of-distribution (OOD) samples, such as individual cells and patients. We conceptualize each cancer type as a distinct source domain, with its cell lines serving as domain-specific samples, and then used adversarial domain generalization to capture essential task-relevant features across multiple source domains. In particular, we are inspired by the observations that an anticancer drug exhibit initial effectiveness, but cancer cells often develop resistance through distinct biological mechanisms. The phenomenon suggests that drug-sensitive cells share common feature, whereas drug-resistant cells exhibit diverse and heterogeneous traits. So, we propose a Fourier asymmetric attention constraint that drives the sensitive samples aggregated into a single compact cluster, while resistant samples dispersed across frequency space. To validate its performance, we firstly evaluate it on bulk RNA-seq and drug response data derived from cell lines, using a leave-one-out validation strategy to assess its generalizability in predicting drug responses for unseen cancer types during training. The results demonstrated that our model achieved superior predictive performance across ten major cancer types. Moreover, we applied the model, trained exclusively on bulk RNA-seq of cell lines, to single-cell and patient-level prediction tasks. The results confirmed it achieved better or comparable performance compared to current state-of-the-art (SOTA) methods. Moreover, our model effectively captured the dynamic transition to resistance when cancer cells subjected to persistent drug exposure. These findings underscore the potential of our method for real-world clinical applications.

## 2 Materials and methods

### 2.1 Data resource and preprocessing

#### 2.1.1 Bulk drug sensitivity

**Figure 1:**
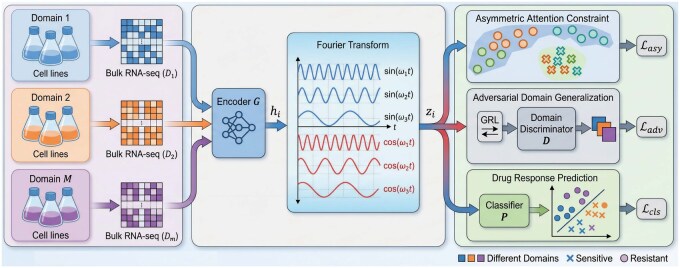
Illustrative diagram of FourierDrug architecture characterized by Fourier transform, asymmetric attention constraint module, with the adversarial domain generalization implemented by gradient reversal layer (GRL).

We regard the bulk RNA-seq data from different cell lines of same cancer type as a source domain, with drug response (sensitive vs resistant) as class labels. The dataset were obtained from the Genomics of Drug Sensitivity in Cancer (GDSC) project (Yang *et al.* 2013), which contained a wealth of data about the responses of 1074 cancer cell lines to 226 therapeutic agents. These cell lines come from >20 cancer types. The drug sensitivity were quantified using the half maximal inhibitory concentration (IC50). Similar to prior studies (He *et al.* 2022, Huang and Liu 2025), we utilized the IC50 values provided by the GDSC to assign drug response labels (sensitive vs. resistant) to cell lines for the drugs of interest. We first identified all cell lines treated by the drug of interest. Next, the cell lines were then ranked based on their IC50 values and categorized into two classes: sensitive (labeled as 1) and resistant (labeled as 0), with the threshold defined as the average IC50 value. It is worth noting that this simple but robust binarization strategy is further validated by the high correlation (ρ= 0.977) between IC50 and AUC values in the GDSC dataset. Unlike quantile-based discretization that discard substantial data, this approach maintains a balanced sample size, ensuring model stability and reducing overfitting risks. Furthermore, distribution analysis of 40 drugs confirms that IC50 values follow an approximately normal distribution (Fig. 1, available as supplementary data at *Bioinformatics* online), supporting the statistical validity of using the mean as a classification threshold.

#### 2.1.2 Single-cell drug response

For performance evaluation, we collected the scRNA-seq data where cells were derived from different cell lines. The scRNA-seq data and single-cell drug response labels were obtained from the National Center for Biotechnology Information’s (NCBI) Gene Expression Omnibus (GEO). Quality control and preprocessing of the scRNA-seq data were performed using the Python package SCANPY (Wolf *et al.* 2018). Specifically, to eliminate lowly expressed genes that may result from noise or background signals, we filtered out cells with fewer than 200 detected genes and genes detected in fewer than 3 cells. To identify and exclude cells that may be contaminated or in an unhealthy state, we also filtered out cell samples with a mitochondrial gene expression ratio exceeding 5%. Besides, because drug-induced changes in gene expression reflect the substantial effect of a drug on cellular phenotype, we selected 3000 highly variable genes from the expression profiles of over 10 000 genes available in the bulk RNA-seq data of cell lines. These selected genes were used as inputs to our model. Note that these genes were selected solely based on the variance of their expression profiles, without utilizing any drug response labels.

#### 2.1.3 Patient-level drug response

The patient expression profiles was retrieved from TCGA repository (https://www.cancer.gov/tcga) (Hutter and Zenklusen 2018), which included bulk RNA-seq data and clinical drug response information across various cancer types. The drug response labels of the TCGA patients were assigned using the similar method to a recent work (Duan *et al.* 2025). Specifically, the responders were those who had a partial or complete response to specific drug treatment, while the non-responders were those who had the progressive clinical disease or stable disease diagnosis. Only the patients received single-drug therapy through the entire duration of treatment were retained, while those treated by drug combinations were excluded.

### 2.2 Problem definition

Assume we have *M* domains $D=\{D_{1},D_{2}\ldots D_{M}\}$, with each domain corresponds to a specific cancer type. Each domain $D_{k}$ comprises the gene expression profiles of $N_{k}$ cell lines regarding to *k*-th cancer type, denoted as $D_{k}=\{X_{1},X_{2},\ldots X_{N_{k}}\}$. For a given drug, the response labels of the $N_{k}$ cell lines in domain $D_{k}$ are represented by $Y_{k}=\{y_{1},y_{2},\ldots ,y_{N_{k}}\}$. Our primary objective is to build a deep learning-based model that accurately map the gene expression profiles of the cell line to their respective drug response labels. Once trained, the model can generalize effectively to predict drug responses in target domains, such as single-cell or patient samples.

### 2.3 Model architecture

The proposed architecture consists of five components: a shared encoder, a Fourier transform module, an asymmetric attention constraint, a classifier, a domain discriminator. As shown in Fig. 1, the expression profiles of cell lines are taken as input to the encoder for feature extraction. Next, Fourier transform is introduced to project the encoded feature to frequency space, in which we impose an asymmetric attention constraint that would aggregate sensitive samples tightly together while dispersing resistant samples. Meanwhile, the domain discriminator aims to distinguish the domain of each sample (i.e. to identify the cancer type from the expression profile). The encoder and domain discriminator are adversarially trained so that the encoder is incentivized to learn features that make it increasingly difficult for the domain discriminator to correctly identify the domains. This adversarial training enables the encoder to capture domain-invariant features pertinent to drug response.

### 2.4 Fourier asymmetric attention constraint

Anticancer drugs are typically designed to target specific molecules or disrupt the biological processes critical for tumor cell proliferation. So, we observed that tumor cells are initially sensitive to some specific drugs, and subsequently develop resistance through diverse biological mechanisms, such as genetic mutations, alterations in cell cycle checkpoints, activation of alternative proliferation signaling pathways, and epigenetic modifications. Correspondingly, we assume that sensitive cells share common pattern in the frequency domain, while resistant cells exhibit diverse heterogeneity. To reflect this observation, we introduce an Fourier asymmetric attention constraint (FAAC) into the domain generalization framework.

Assume that the feature space is $R^{d}$, we construct a set of function bases isomorphic to it, mapping the feature space into frequency space. Formally, in the continuous domain over the interval [−T,T], the sine and cosine functions form a complete orthogonal basis $\Phi =\{\phi_{k}(x),\psi_{k}(x)\}$, defined as:


(1) $$\phi_{k}(x)=\text{cos}\;\left(\frac{2\pi kx}{T}\right),\;\psi_{k}(x)=\text{sin}\;\left(\frac{2\pi kx}{T}\right),\;k\in N.$$

A function f(x) defined on [−T,T] can be expanded using this basis as a Fourier series:


(2) $$f(x)=\sum_{k=1}^{\infty }\left(a_{k}\phi_{k}(x)+b_{k}\psi_{k}(x)\right),$$

where $a_{k}$ and $b_{k}$ are Fourier coefficients. Because the encoded features are represented as discrete vectors, we discretize the interval [−T,T] into *d* uniformly spaced sampling points $\left\{x_{1},x_{2},\ldots ,x_{d}\right\}$. We then construct the discrete orthogonal basis vectors by sampling the sine and cosine functions at these points:


(3) $$\phi_{k}={\left[\text{cos}\;\left(\frac{2\pi kx_{1}}{T}\right),\ldots ,\;\text{cos}\;\left(\frac{2\pi kx_{d}}{T}\right)\right]}^{⊤},$$

(4) $$\psi_{k}={\left[\text{sin}\;\left(\frac{2\pi kx_{1}}{T}\right),\ldots ,\;\text{sin}\;\left(\frac{2\pi kx_{d}}{T}\right)\right]}^{⊤}.$$

For each feature vector $h_{i}\in R^{d}$ extracted by encoder *G*, we project it onto the discrete orthogonal basis to obtain its representation in the frequency domain:


(5) $$z_{i}≜F(h_{i})=\sum_{k=1}^{d/2}\langle h_{i},\phi_{k}\rangle \phi_{k}+\langle h_{i},\psi_{k}\rangle \psi_{k},$$

where F() represents Fourier transform, 〈·,·〉 denotes the Euclidean inner product. Note that $z_{i}$ does not represent the Fourier expansion, it instead corresponds to a set of Fourier coefficients obtained from the Fourier expansion. As a result, the transformation maps the latent representation of expression profiles into frequency domain, enabling explicit control over frequency spectrum. To enhance the alignment of semantically similar samples in the frequency domain, we introduce an asymmetric constraint based on the Fourier-transformed representations. Specifically, given a positive sample $z_{i}$ as an anchor, we aim to maximize the cosine similarity between the anchor and positive samples, while minimizing the cosine similarity between the anchor and negative samples. Importantly, we do *not* impose any constraint between the negative samples themselves. This leads to the following asymmetric loss function:


(6) $$L_{\text{asy}}=-\frac{1}{\left|P_{i}\right|}\sum_{j\in P_{i}}\frac{z_{i}^{⊤}z_{j}}{\left\|z_{i}\|\cdot \|z_{j}\right\|}+\frac{1}{\left|N_{i}\right|}\sum_{k\in N_{i}}\frac{z_{i}^{⊤}z_{k}}{\left\|z_{i}\|\cdot \|z_{k}\right\|},$$

where $P_{i}$ represents the set of positive samples (drug-sensitive), and $N_{i}$ represents the set of negative samples (drug-resistant).

We further illustrate that the asymmetric constraint mathematically correlates to self-attention mechanism. In standard Transformer architectures (Vaswani *et al.* 2017), self-attention weight is computed via scaled dot-product similarity between the query and key embedding:


(7) $$\alpha_{i,j}=\frac{q_{i}^{⊤}k_{j}}{\sqrt{d_{k}}}.$$

Since $z_{i}^{⊤}z_{j}=\|z_{i}\|\cdot \|z_{j}\|\cdot \;\text{cos}(z_{i},z_{j})$, we can interpret the cosine similarity in frequency domain as a **normalized form** of the attention dot-product, thereby capturing angular correlations between frequency components of the input features. Furthermore, according to the Convolution Theorem, i.e. the element-wise multiplication in the frequency domain corresponds to convolution in the time domain, we can interpret $z_{i}^{⊤}z_{j}$ as an approximation of the operation between $x_{i}$ and $x_{j}$ through continuous-space convolution. Unlike the standard self-attention mechanism, our approach achieves global attention associations without increasing the number of parameters. Our experiments demonstrate that this approach not only enhances the model’s generalizability but also effectively mitigates overfitting (See Section 3).

### 2.5 Adversarial domain generalization

The objective of domain generalization is to learn domain-invariant features relevant to the prediction task, while eliminating domain-specific information. For this purpose, we introduce a domain discriminator designed to classify the domain of each input sample. The encoder and domain discriminator are trained in an adversarial manner, with the encoder learning to extract features that prevent the discriminator from accurately identifying the domain. This adversarial training process encourages the encoder to capture features that are both predictive of drug response and consistent across all domains. Denote by $L_{\mathrm{adv}}$ the cross entropy loss for domain discrimination, we have


(8) $$\underset{D}{\min}\underset{G}{\max}L_{\text{adv}}=-\sum_{k=1}^{M}\sum_{i=1}^{N_{k}}d_{i}^{\left(k\right)}\;\text{log}\;D(z_{i}^{\left(k\right)})$$

in which $d_{i}^{\left(k\right)}$ is the true domain label of the input sample $X_{i}^{\left(k\right)}$ from domain $D_{k}$, $D(z_{i}^{\left(k\right)})$ represents the domain label predicted by the domain discriminator *D* given the representation $z_{i}^{\left(k\right)}$ obtained from Equation (5). The domain discriminator aims to minimize the loss, whereas the encoder strives to maximize it. In our practice, we use the Gradient Reversal Layer (GRL) to implement the adversarial training between the encoder and the domain discriminator.

### 2.6 Drug response predictor

The drug response labels of cell lines subjected to specific drug exposure is used to train the classifier. It takes as input the Fourier-transform feature to predict the response labels. We use cross-entropy as the classification loss, with the loss function $L_{\mathrm{cls}}$ defined as:


(9) $$\underset{P}{\min}L_{\text{cls}}=-\sum_{k=1}^{M}\sum_{i=1}^{N_{k}}y_{i}^{\left(k\right)}\;\text{log}(P(z_{i}^{\left(k\right)}))+(1-y_{i}^{\left(k\right)})\;\text{log}(1-P(z_{i}^{\left(k\right)}))$$

in which $P(z_{i}^{\left(k\right)})$ represents the predicted response label by the predictor, and $y_{i}^{\left(k\right)}$ is the true response label.

Finally, the total loss is defined as below:


(10) $$L=L_{\text{adv}}+\lambda_{1}L_{\text{asy}}+\lambda_{2}L_{\text{cls}}$$

where $\lambda_{1}$ and $\lambda_{2}$ are the tradeoff parameters.

In our practice, the encoder are realized using fully connected feed-forward networks with rectified linear unit (ReLU) activation function. It consist of only two feed-forward layers with sizes of 1024 and 740, respectively. Each feed-forward layer is followed by a batch normalization layer, and a dropout layer with the dropout probability set to 0.1. The learning rate is set to 8e−5. Our model was implemented in PyTorch 3.10, and all experiments were conducted on a CentOS Linux 8.2.2004 (Core) system, equipped with a GeForce RTX 4090 GPU and 128 GB memory. During the model training and cross-validation stage, these loss terms were appropriately weighted.

## 3 Results

### 3.1 Drug response prediction for unseen cancer type

We first evaluated the performance of FourierDrug in predicting drug response for unseen cancer types during training. For objective evaluation, all cell lines of a specific cancer type were designated as the test set, while cell lines from other cancer types were used for training. This leave-one-out approach enabled us to evaluate the performance of FourierDrug on unseen cancer types for a specific drug. Since the expression profiles of cell lines from the same cancer type are generally similar, we excluded all cell lines of a given cancer type from the training set, and thus effectively eliminates the risk of data leakage and enables us to rigorously test whether the model can capture drug response-relevant features from the expression profiles of other cancer types. As a result, the leave-one-out test set included ten cancer types: Lung Adenocarcinoma (LUAD), Small Cell Lung Cancer (SCLC), Breast Cancer (BRCA), Colorectal Adenocarcinoma (COREAD), Head and Neck Squamous Cell Carcinoma (HNSC), Ovarian Cancer (OV), Neuroblastoma (NB), Pancreatic Adenocarcinoma (PAAD), Acute Myeloid Leukemia (LAML), and Mesothelioma (MESO). Our evaluation considered five distinct drugs: Afatinib, AR-42, Docetaxel, Etoposide, and PLX4720. These drugs represent a diverse array of therapeutic classes, including chemotherapy agents, targeted therapies, and broad-spectrum inhibitors. It is worth noting that the model was trained in a drug-wise manner, based on the rationale that different drugs may exhibit distinct mechanisms of action.

**Figure 2:**
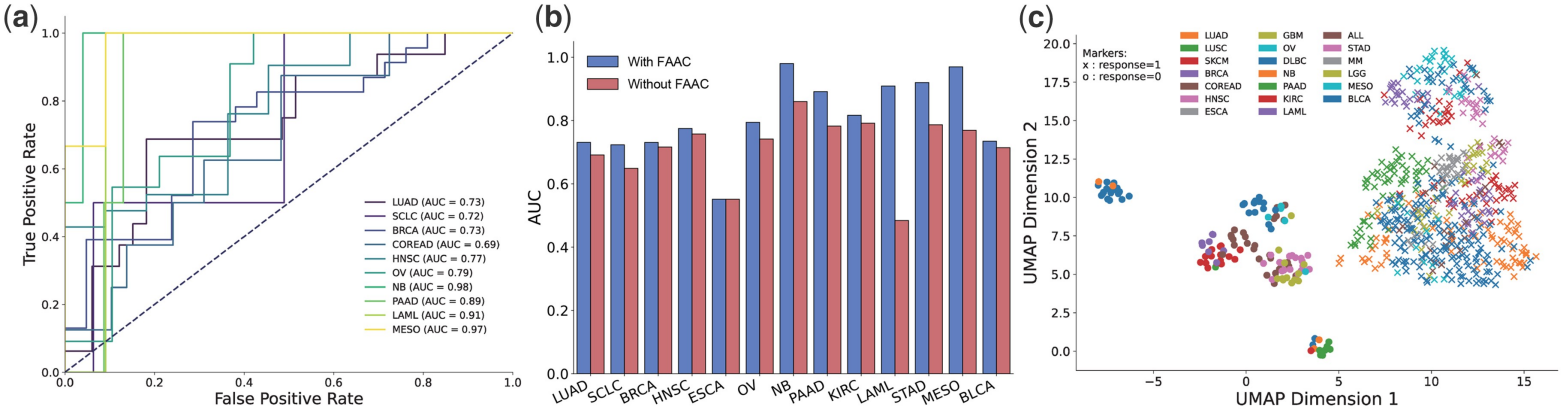
Performance evaluation of FourierDrug in predicting bulk drug responses for hold-out cancer types. (a) ROC curves for ten hold-out cancer types. (b) AUROC values achieved by our model with and without Fourier asymmetric attention constraint (FAAC) module. (c) UMAP plots of the learned features of 20 cancer types included in the training set.

**Figure 3:**
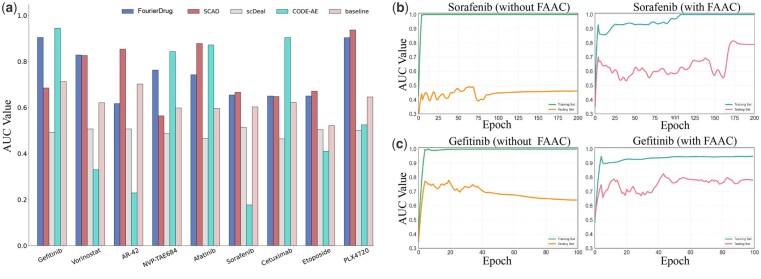
Performance evaluation of FourierDrug trained on bulk RNA-seq in predicting single-cell drug response. (a) Performance comparison with baseline and SCAD for nine distinct drugs. (b) and (c) AUROC values achieved by FourierDrug for Sorafenib (b) and Gefitinib (c) with respect to training epochs with and without Fourier asymmetric attention constraint (FAAC) module.

The experimental results demonstrated that our model achieved superior performance in predicting drug responses across ten major cancer types (Fig. 2a), with particularly high AUROC values exceeding 0.9 in the cancers such as neuroblastoma (NB), mesothelioma (MESO), and acute myeloid leukemia (LAML). Next, we validated the effectiveness of the latent independent projection in improving performance. As illustrated in Fig. 2b, inclusion of this module led to remarkably improved model performance. To further validate that the encoder captured the discriminative feature related to drug response, we used UMAP to project the learned features into the 2D space. It can be found that the sensitive and resistant sample were clearly separated (Fig. 2c). Particularly, the sensitive samples gathered into a cluster closely, whereas the resistant samples dispersed into multiple separate clusters. This results strongly verified the validity of the asymmetric attention constraint. To provide a quantitative assessment of the effect of the asymmetric constraint, we computed the Average Silhouette Width (ASW) in both the original expression profiles and the Fourier-transformed feature space. The results show that, after applying the FAAC constraint, the ASW of the frequency-domain features improved substantially from 0.0239 to 0.4926. This quantitative improvement demonstrates that the model effectively separates sensitive and resistant samples, as illustrated in Fig. 2, available as supplementary data at *Bioinformatics* online. Moreover, we performed regression analysis between the learned features and the real IC50 values, comparing them to PCA features that accounted for 95% variance across all genes. The results indicated that the features extracted by the encoder provided a better fit to the IC50 values. In contrast, we plotted the UMAP of the original bulk RNA-seq data of the cell lines, and found that the sensitive and resistant samples belonging to a same cancer type are intermixed and not easily distinguishable (see Fig. 3, available as supplementary data at *Bioinformatics* online). These findings confirmed that our model successfully captured drug response-related features from gene expression profiles, rather than simply memorizing drug response labels, thereby enabling it to predict the drug responses for unseen cancer types during training.

### 3.2 Accurate prediction of single-cell drug response

The inherent heterogeneity of tumors often leads to significant variability in gene expression profiles of individual cancer cells within a tumor. Meanwhile, the noise present in single-cell RNA sequencing (scRNA-seq) data further complicates this issue, leading to data distributions that differ substantially from the bulk RNA-seq data used during model training. To assess the generalizability of our proposed method, we systematically evaluated its performance in predicting drug responses at the single-cell level, where individual cells are treated as samples from a new domain distinct from the training data.

The single-cell drug response datasets used for performance evaluation comprised both pre-treatment scRNA-seq data from CCLE (Barretina *et al.* 2012) and post-treatment scRNA-seq data from GEO repository (accession numbers: GSE149215 and GSE108383). The pre-treatment dataset included the expression profiles and drug response labels of JUH006 cell line to the treatment of three distinct drugs (Gefitinib, Vorinostat and AR-42), as well as the data of SCC47 cell line treated with other four distinct drugs (NVP-TAE684, Afatinib, Sorafenib, Cetuximab). The post-treatment datasets contained the expression profiles and drug response labels of PC9 cell line following Etoposide treatment (Aissa *et al.* 2021), as well as the A375 and 451Lu cell line treated with PLX4720 (Ho *et al.* 2018). For performance evaluation on scRNA-seq data, we selected a subset of highly variable genes that exhibited the most significant differences in expression levels across both bulk and single-cell RNA-seq data, and used these genes as inputs into our model. The details about the single-cell drug response datasets are listed in Table 1.

**Table 1:** Detail of single-cell drug response datasets for FourierDrug performance evaluation.

Source	Drug	Cell line	No. of Res.	No. of Sen.	No. of gene
GSE149215	Etoposide	PC9	764	629	9738
GSE108383	PLX4720	A375	46	62	11 937
CCLE	Geftinib	JUH006	33	33	10 610
CCLE	Vorinostat	JUH006	33	33	10 610
CCLE	AR-42	JUH006	33	33	10 610
CCLE	NVP-TAE684	SCC47	60	60	10 684
CCLE	Afatinib	SCC47	60	60	10 684
CCLE	Sorafenib	SCC47	60	60	10 684
CCLE	Cetuximab	SCC47	60	60	10 684

To benchmark performance, we build a baseline model composed of only three fully connected layers, with an input dimension of 740, a first hidden layer of 128 units, a second hidden layer of 64 units, and a single output unit for the classification task (i.e. drug response labels). ReLU activation functions are applied to all layers. The baseline model was trained on the GDSC dataset and then directly applied to the single-cell datasets. Meanwhile, we conducted performance comparison to three state-of-the-art methods: SCAD (Zheng *et al.* 2023), scDEAL (Chen *et al.* 2022), and CODE-AE (He *et al.* 2022). SCAD and scDEAL are domain adaptation-based methods designed for predicting single-cell drug responses, while CODE-AE leverages feature disentanglement to extract common feature between source and target domains. All competing methods were trained and tested on our established benchmark dataset, with all methods running on the same workstation. The experimental results showed that our method remarkably outperformed the baseline model across all drugs (Fig. 3a). Particularly, compared to SCAD that used SMOTE sampling for class balance and top 4k highly variable gene as input (smote_tp4k), our method achieved better or comparable performance for most drugs. The performance of CODE-AE is highly unstable. While it achieves outstanding results for specific drugs, such as Gefitinib and Cetuximab, it performs poorly for other drugs, including Vorinostat, AR-42, Sorafenib, and Etoposide. Notably, these competing methods utilized the scRNA-seq data as target domain during the training stage, while our method never used scRNA-seq data during training. This highlights the ability of our method to extract generalizable features related to drug response from gene expression profiles across diverse source domains via domain generalization. So, it achieves robust performance without the need for target domain data during training, exemplifying a “train once, adapt anywhere” framework.

**Figure 4:**
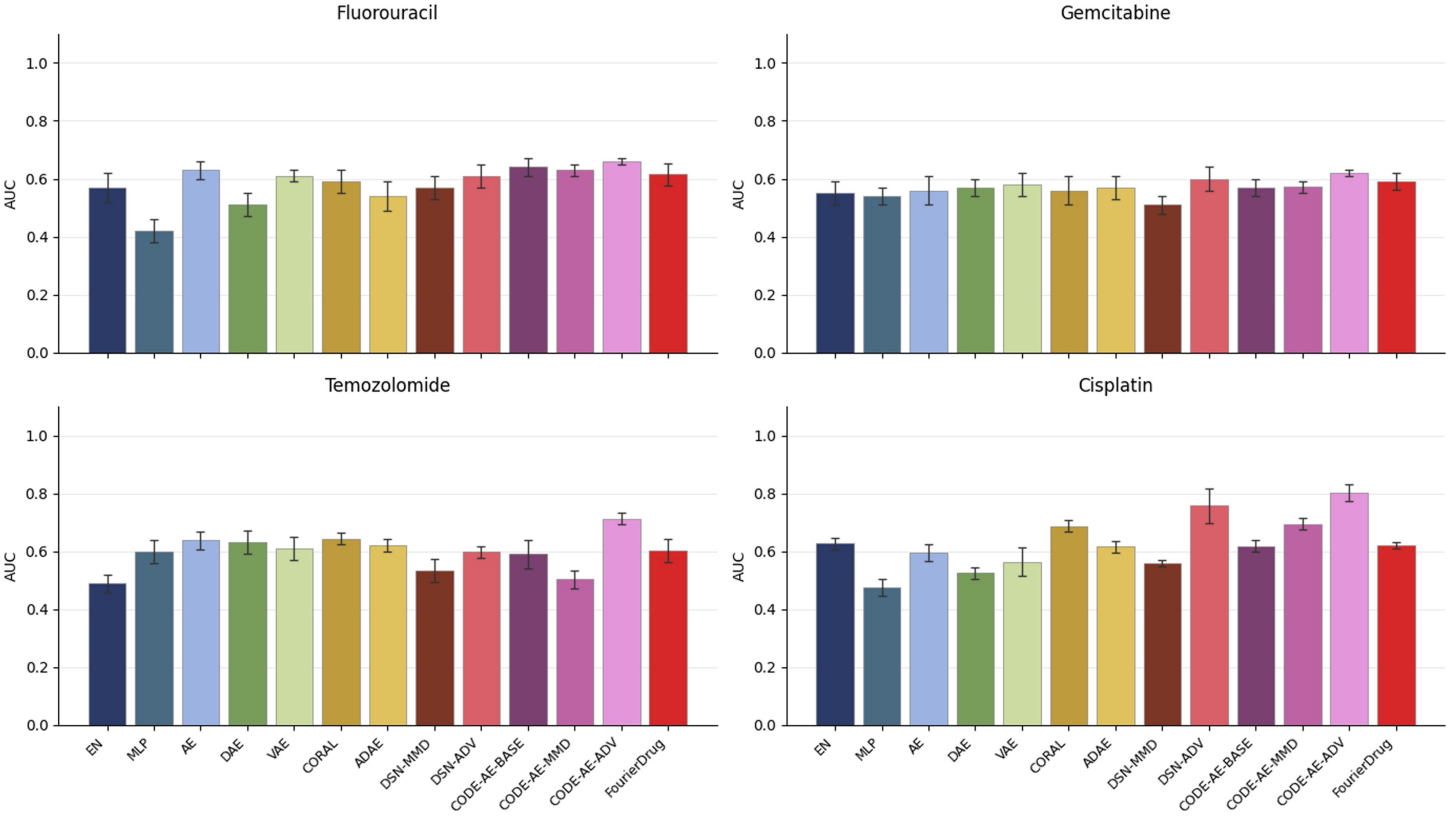
Performance comparison of FourierDrug with 12 existing methods on the prediction of TCGA patient drug response.

Furthermore, we assess the effectiveness of the FAAC module in improving model generalizability. By comparing the performance with and without FAAC module, we evaluate its impact on predicting single-cell response for each drug. Due to space limitations, we only present the AUROC values by our method for two drugs, Sorafenib and Gefitinib, with the increasing of training epochs (Fig. 3b and c). We further evaluated the performance of FourierDrug on additional drugs (see Fig. 4, available as supplementary data at *Bioinformatics* online). The results demonstrate that the framework maintains robust predictive performance across different drug treatments, and validated that incorporating the FAAC module effectively prevents overfitting, thereby improving our model’s generalizability to out-of-distribution data.

### 3.3 High generalizability to patient drug response

For further evaluation, we applied FourierDrug to predict clinical drug responses in patients. *In vivo* drug response prediction poses significant challenges due to the influence of many biochemical factors, making it inherently more difficult than *in vitro* predictions on cell lines. This experimental setting provides a more stringent evaluation of the model’s generalizability.

The expression profiles and clinical metadata of patients were obtained from the TCGA repository (Hutter and Zenklusen 2018). The patients treated with one of four drugs—Fluorouracil, Gemcitabine, Temozolomide, or Cisplatin—were selected for analysis. The four drugs were chosen due to the relatively large number of patients subjected to the treatments of these drugs, enabling objective and reliable performance evaluations. Patients exhibiting a complete response or partial response were labeled as sensitive, while those with clinically progressive or stable disease were labeled as resistant. From the patient expression profiles, 3000 differentially expressed genes (DEGs) were identified based on a significance threshold of *P*-value≤.05. The DEGs were then intersected with gene expression data from the GDSC dataset, and the overlapping genes were subsequently used as inputs for our model. Especially, the overlapped genes were selected solely based on the variance of their expression profiles, without utilizing any patient drug response labels. This procedure is part of the “test-time preprocessing.”

For performance evaluation, we compared it with 12 previously published methods. These methods include conventional machine learning classifiers (e.g. MLP, EN) and deep learning models (e.g. AE, VAE, DAE), as well as various domain adaptation methods (e.g. ADAE, CORAL, DSN variants, CODE-AE variants). As illustrated in Fig. 4, our model demonstrated superior performance across all drugs except Cisplatin, where it marginally underperformed relative to CODE-AE-ADV (He *et al.* 2022) and DSN-ADV (Bousmalis *et al.* 2016) but outperformed other methods.

**Figure 5:**
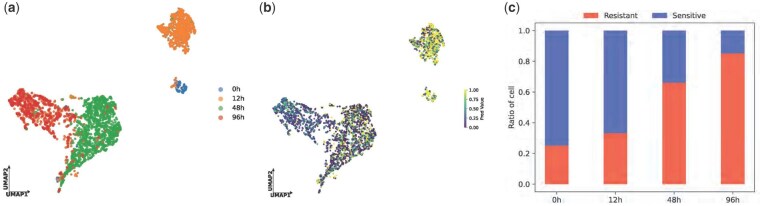
Accurate prediction of dynamic transition to resistance of MCF7 cells in response to Bortezomib treatment at four time points. (a) UMAP visualization of scRNA-seq data of MCF7 cells colored by different time points. (b) UMAP visualization of MCF7 cells colored by predicted sensitivity scores. (c) Time-dependent decline in the proportion of drug-sensitive cells with increasing duration of exposure.

Furthermore, we conducted ablation studies on four additional drugs—Docetaxel, Paclitaxel, Sorafenib, and Vinorelbine—to evaluate the contribution of the latent independent projection module. The results verified that the inclusion of this module brought substantial performance gains, exceeding 10% across all four drugs (Fig. 5, available as supplementary data at *Bioinformatics* online). The most pronounced improvement was observed for Sorafenib, with an AUROC value increase by over 30%.

### 3.4 Predicting single-cell dynamic transition toward drug resistance

Cancer cells are known to rapidly evolve under therapeutic pressure, often developing resistance to specific drugs. To evaluate the generalizability of FourierDrug, we investigated its ability to predict temporally dynamic changes in individual cell states during prolonged chemotherapy exposure. For this analysis, we utilized the dataset published by Ben-David *et al.* (2018), comprising 7440 single-cell clones derived from the MCF7 cell line. These clones were exposed to 500 nM Bortezomib for two days and underwent single-cell sequencing at distinct time points: prior to treatment (t0, n=160), 12 h post-treatment initiation (t12, n=994), 48 h post-exposure (t48, *n*=1 623), and 96 h following drug washout and recovery (t96, n=963). UMAP visualization revealed four transcriptionally distinct clusters corresponding to the time points analyzed (Fig. 5a), indicating significant transcriptional divergence driven by drug exposure.

To assess whether FourierDrug could accurately capture the state transitions of MCF7 cells under sustained Bortezomib exposure, we applied it to predict drug response states and analyzed the predicted sensitivity scores of individual cells (Fig. 5b). The predictions showed that nearly all MCF7 cells were highly sensitive to Bortezomib at baseline (t0). However, with increasing exposure duration, the proportion of sensitive cells gradually declined, accompanied by a rise in resistant cell populations. By 96 h post-treatment (t96), the cell population exhibited the highest fraction of resistant cells (Fig. 5c). These results closely aligned with observations from prior studies (Pellecchia *et al.* 2023), further substantiating the model’s predictive accuracy. Our findings demonstrate that FourierDrug effectively captures the temporal dynamics of cellular responses to drug treatment across multiple time points, highlighting its potential as a robust tool for modeling drug-induced phenotypic evolution in cancer cells.

## 4 Discussion and conclusion

In this study, we propose a novel domain generalization framework designed to predict drug responses without access to target-domain data during training. Our empirical experiments confirmed that our method outperforms current state-of-the-art methods in predicting drug responses at both single-cell and patient levels, demonstrating its potential for clinical applications. The proposed framework represents a significant advancement in computational drug response prediction, addressing a critical translational challenge: bridging preclinical models and clinical heterogeneity through domain-invariant representation learning. Its innovation lies in the integration of adversarial domain generalization and biologically inspired Fourier asymmetric attention constraints. This combination enables robust generalization across unseen cancer types and diverse data modalities (e.g. bulk RNA-seq to single-cell or patient data). By training on multi-source preclinical data while mitigating cancer type-specific biases, the framework eliminates reliance on target-domain data during training—a limitation of traditional domain adaptation approaches. This capability aligns with real-world clinical scenarios, where newly diagnosed patient data are unavailable during model development, verifying FourierDrug as a powerful tool for bridging preclinical research and clinical application.

The strength of FourierDrug lies in its frequency-based asymmetric constraint that encodes the biological reality that drug-sensitive cells often share common vulnerabilities, whereas resistance mechanisms exhibit context-dependent heterogeneity. This feature alignment enhances prediction accuracy while providing interpretability, as latent clusters can be mapped to known resistance pathways. This represents a distinct advantage over “black-box” models that lack mechanistic insights. Additionally, the Fourier asymmetric attention module addresses the challenges of high-dimensional data by enforcing non-redundant feature extraction through trigonometric orthogonal bases. This approach is methodologically unique compared to soft orthogonality constraints, which are prone to optimization instability. Validation across multiple scales and modalities highlights the framework’s versatility. Its strong performance on bulk RNA-seq data (cell lines), single-cell RNA-seq (scRNA-seq), and clinical datasets demonstrates unprecedented cross-domain generalizability. So, we conclude that the parameter-free, global “attention” mechanism mirrors the effects of self-attention yet adds no extra trainable parameters, yielding more efficient, interpretable feature learning that reflects true biological diversity.

Despite these advancements, the framework’s reliance on transcriptomic data does not account for other determinants of drug response, such as somatic mutations and copy number variations (CNVs). Future iterations of the framework could adopt a multi-omics integration strategy to capture the interplay between genomic alterations and drug sensitivity. Another limitation is the binary simplification of drug response (sensitive/resistant). While the asymmetric attention constraint accounts for resistance heterogeneity, it neglects intermediate phenotypes (e.g. partial sensitivity) and dose-dependent effects—a gap highlighted by pharmacological databases such as CancerDR (Kumar *et al.* 2013), which incorporate IC50 gradients and mutation-specific drug responses. Incorporating continuous response metrics and probabilistic clustering could better reflect clinical realities, particularly for therapies with narrow therapeutic windows. Finally, FourierDrug is primarily designed for small-molecule drugs characterized by well-defined IC50 dose-response curves. The therapies such as immunotherapy rely heavily on the complex dynamics of the tumor immune microenvironment, which involves multi-layered spatial organization and intricate cell-cell interactions. We therefore acknowledge that our current single-modality gene expression modeling alone may be insufficient to fully capture the complexity of treatment responses.

In summary, the FourierDrug framework establishes a novel paradigm for predicting drug responses without needing target-domain data during training. By using Fourier transforms and asymmetric attention constraints, the approach aggregates sensitive samples and disperses resistant ones, achieving robust feature extraction. Validated on bulk RNA-seq, single-cell, and patient-level data, FourierDrug outperformed state-of-the-art methods, demonstrating exceptional cross-domain generalizability.

## Supplementary Material

btag276_Supplementary_Data

## Data Availability

The public datasets analyzed during the current study are available in the TCGA repository (https://portal.gdc.cancer.gov/), GDSC database (https://www.cancerrxgene.org/), and CCLE portal (https://depmap.org/portal/ccle/). The source code, trained models, and all supplementary experimental results (including multi-seed and stochastic 100-run simulation data) generated during this study are openly available in the GitHub repository at https://github.com/hliulab/FourierDrug.
