# Supplementary material for: FourierDrug: a domain generalization framework for robust drug response prediction via frequency-space asymmetric attention
Source: Bioinformatics. 2026 May 5;42(6):btag276. doi: 10.1093/bioinformatics/btag276 (PMC13271248; doi:10.1093/bioinformatics/btag276)
Supplement: btag276_Supplementary_Data [file btag276_supplementary_data.pdf]

Supplementary file of FourierDrug

Ran Song, Yinpu Bai, Judong Luo*, Hui Liu*

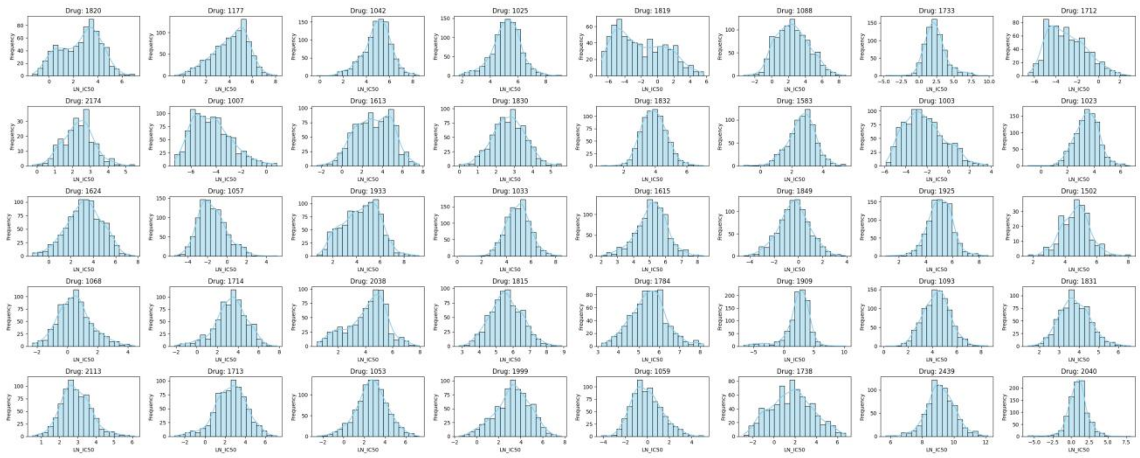

Figure S1 Distributions of IC50 values regarding 40 drugs randomly selected from GDSC dataset

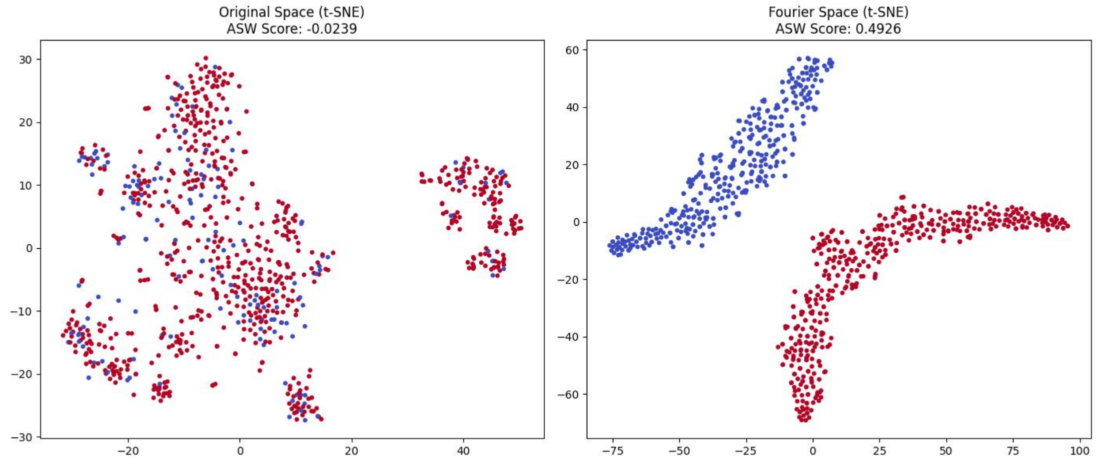

Figure S2 Visualization of original expression profiles and the Fourier-transformed features

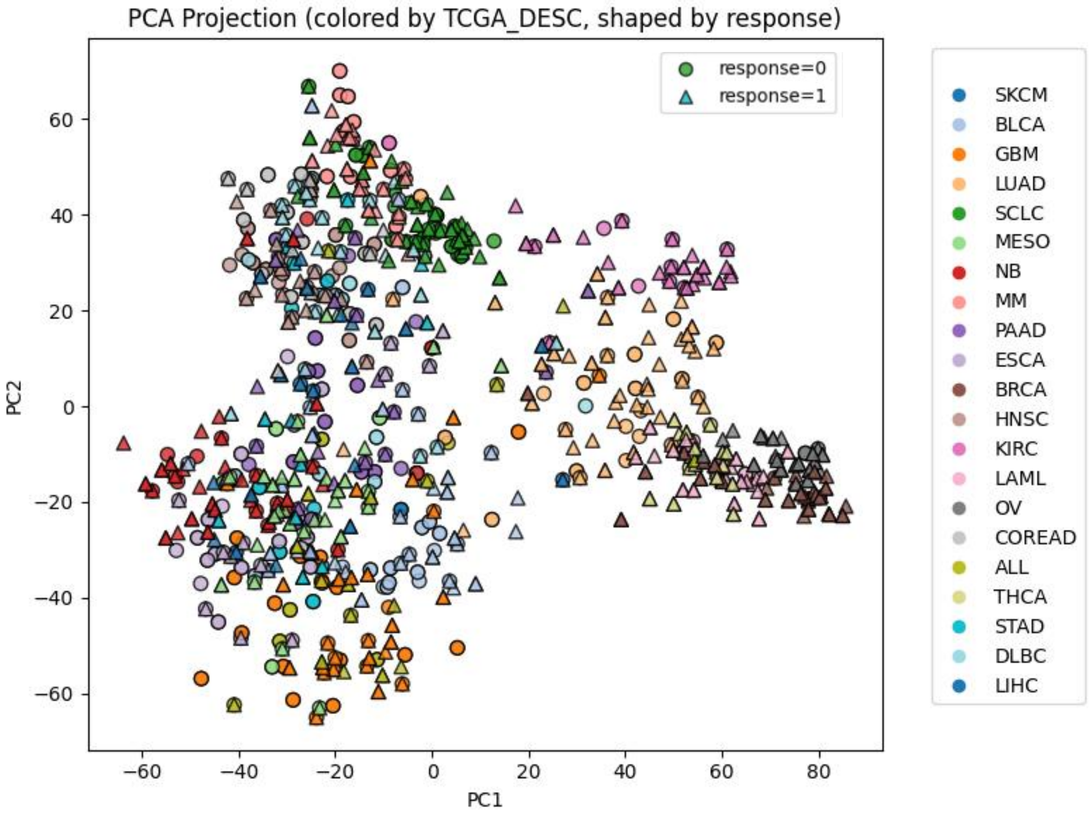

Figure S1. UMAP visualization of the original expression profiles of cell lines in GDSC dataset.

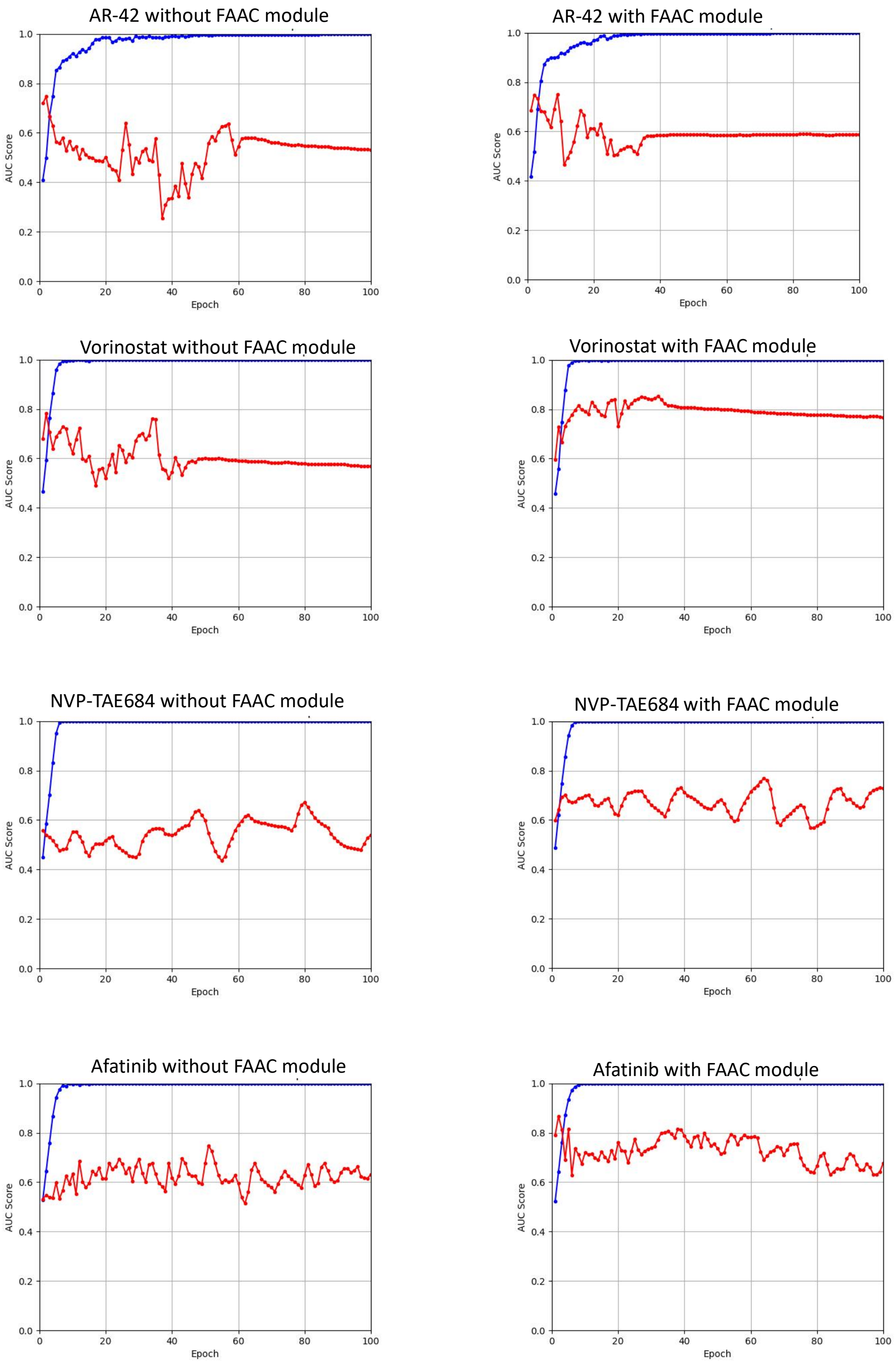
AR-42 without FAAC module
AR-42 with FAAC module
Vorinostat without FAAC module
Vorinostat with FAAC module
NVP-TAE684 without FAAC module
NVP-TAE684 with FAAC module
Afatinib without FAAC module
Afatinib with FAAC module
AUC Score
Epoch
0.0
0.2
0.4
0.6
0.8
1.0
0
20
40
60
80
100

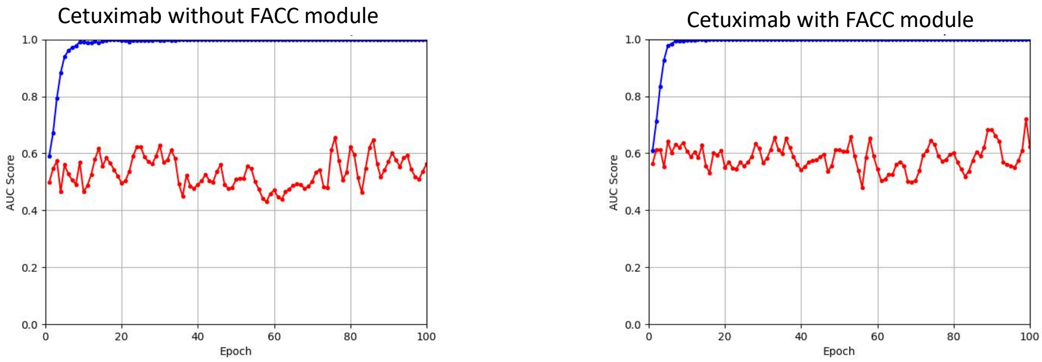

Figure S4 AUROC curves with respect to training epochs of FourierDrug with and without FAAC module (left: without FAAC, right: with FAAC).

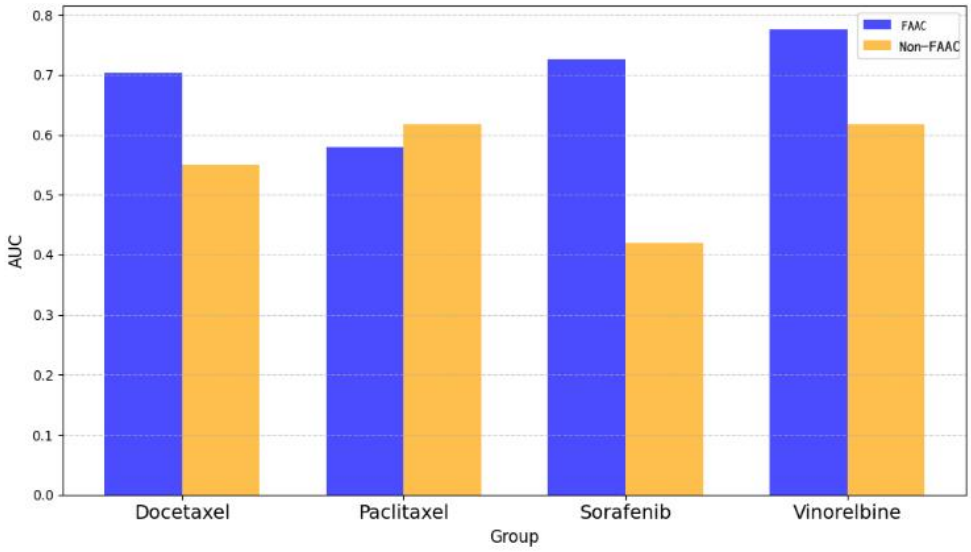

Figure S5 AUROC values achieved by FourierDrug with and without FAAC module.
